# Human Figure Drawings in Children with Autism Spectrum Disorders: A Possible Window on the Inner or the Outer World

**DOI:** 10.3390/brainsci10060398

**Published:** 2020-06-23

**Authors:** Pamela Papangelo, Martina Pinzino, Susanna Pelagatti, Maddalena Fabbri-Destro, Antonio Narzisi

**Affiliations:** 1 Institute of Neuroscience, National Research Council (CNR), Via Volturno 39/E, 43125 Parma, Italy; pamela.papangelo@gmail.com (P.P.); martinapinzino@outlook.it (M.P.); 2 Department of Informatics, University of Pisa, 56126 Pisa, Italy; susanna.pelagatti@gmail.com; 3 IRCCS Stella Maris Foundation, 56126 Pisa, Italy; antonio.narzisi@fsm.unipi.it

**Keywords:** human figure drawings, Draw-a-Man, drawings maturity, autism spectrum disorders, social perception

## Abstract

Background: Tests based on human figure drawings (HFD) have captured the attention of clinicians and psychologists for a long time. The aim of the present study was to evaluate the performance of HFD of children with autism spectrum disorders (ASDs) relative to typically developing (TD) controls. Methods: All children were asked to draw three human figures (man, woman, self-portrait) and were evaluated with a neuropsychological battery. HFD were scored according to the Maturity Scale, and correlative approaches testing maturity against neuropsychological scores were applied. Results: ASDs presented marked deficits in maturity. No significant correlation emerged for both groups between maturity and the theory of mind test. On the contrary, positive and significant correlations between maturity and the affect recognition test (AR) were found, with group-specific patterns. In TD, this result regarded drawings of others, but not self-portraits, while an opposite pattern emerged for ASD, whose sole maturity in self-portraits significantly correlated with the AR scores. Conclusion: These findings suggest that the use of HFD tests with individuals with autism may not be used in clinical practices. However, in basic research, HFDs could be used to highlight dependencies between drawing performance and neuropsychological features, thus possibly providing hints on the functioning of autism.

## 1. Introduction

Children have been using drawings to express themselves since ancient times and this topic has captured the interest of scientists since the late 19th century. Indeed, by analyzing the presence/absence of graphical aspects like details, colors, proportions, and shapes, it is possible to trace a developmental maturation trajectory based on children’s drawings [1]. Historically, the Draw-a-Man test (DAMT) developed by Goodenough [2] represents the first systematic scoring system for children’s drawings, devised with the intent to provide a surrogate measure of children’s intelligence. Scores were initially based on the number of details and the accuracy of placement of each body part. DAMT was later revised by Harris [3], who proposed that children should be asked to draw not just one but three human figures: a generic man, a generic woman, and a self-portrait. The scoring system for the maturity estimation was updated, accounting also for the precision of details, and proportions.

Since these initial pioneering studies, several revisions and applications have been conducted to enhance the utility of DAMT, and more generally of human figure drawing (HFD) tests, in virtue of their versatility and usability also with children with limited attention span and language difficulties. There is no exception for the use of the HFD in the field of autism.

As far as autism spectrum disorders (ASDs) drawings are concerned, children with autism were reported to have an unusual drawing ability, far beyond their general intelligence level [4,5,6], and drawing skills not impaired relative to age-matched typical peers [7,8]. However, Lee and Hobson [9] reported slightly lower global scores in HFD for children with ASD relative to children with learning difficulties, whereas the same pattern did not emerge across groups in drawings of houses. Similarly, Lim and Slaughter [10] indicated that in HFD, children with autism are generally less sophisticated and detailed than children without autism. Given this controversial pattern of results, whether children with autism differ from age-matched TD peers is still a matter of debate, leaving open the question whether human figure drawings could be useful in the clinical practice of autism. Indeed, while international guidelines [11] excluded tests based on drawings from the batteries used for the diagnosis of ASDs, it is possible that the maturity score covaries in association with other neuropsychological indices not easily obtainable in children with ASDs.

Autism spectrum disorders (ASDs) are a severe multifactorial disorder characterized by an umbrella of peculiarities in the areas of social communication, restricted interests, and repetitive behaviors [12]. The incidence of ASDs is worldwide and recent epidemiological data estimated it to be higher than 1/100 [13]. ASDs vary greatly in the severity of their socio-communicative impairments [14] as well as in cognitive and language development.

Many of the social interpersonal difficulties in ASD derive to some extent from weaknesses in the children’s social perception, which in turn relies on both cognitive [15] and motor processes [16]. Social perception refers to children’s ability to represent and understand others, their mental states, emotions, and beliefs. Concerning theory of mind (ToM), several studies demonstrated that individuals with ASDs have performance lower than individuals with typical development [17]. These deficits are reported using different versions of ToM tasks, including those examining false beliefs [18], cartoon animations [19], or inferences of mental states from photographs [20].

A key aspect of social perception in ASDs pertains to emotion and affect recognition, yet inconsistent findings have been reported so far. Indeed, while Kuusikko et al. [21] and Krebs et al. [22] pointed to an impairment of children with ASDs in emotion recognition, other studies [23,24,25] failed to report such a deficit. To date, no evidence has been provided about the possible relationship between social perception scores and performance in human figure drawing tests.

Starting from these premises, in the present study we evaluated the performance of the Draw-a-Man test in a group of children with ASDs, and compared them against a group of age-matched typically developing controls. In particular, we tested two hypotheses: (1) ASDs have lower maturity scores in DAMT, and (2) performance at DAMT differs according to the subjects to-be-depicted (self vs. representation of others). The value of such an investigation is that if a difference is highlighted (i.e., ASDs present lower scores relative to TD), tests based on human figure drawings should not be considered in ASDs as indexing maturity, unless a proper normative ASD population is acquired. In addition to factorial contrasts, the clinical value of the HFD test could be further characterized by correlative approaches testing maturity against neuropsychological scores most common in the clinical practice of autism.

## 2. Materials and Methods

### 2.1. Participants

Twenty-one typically developing boys (TD, age M = 8.7, SD = 1.8) and 22 boys with autism spectrum disorders (ASDs, age M = 9.2, SD = 1.7) were included in the study. Groups resulted matched for chronological age (*p* = 0.30) and nonverbal IQ assessed by Raven’s Colored Progressive Matrices [26] (*p* = 0.72).

Concerning the ASD group, inclusion criteria were: (a) diagnosis of autism spectrum disorder according to DSM-V/ICD-10 criteria and certified by the Italian Mental Health System (acknowledgement of handicap through Italian law n. 104/1992); (b) age 6–12 years; (c) non-verbal intelligence quotient within the 90–130 range; (d) capacity to adhere to experimental procedures; and (e) lack of comorbidities according to their medical records. Exclusion criteria consisted of (a) presence or history of any other axis I mental disorder; or (b) history of traumatic brain injury or any other neurological disorder as by their medical record. Participants with ASDs were all children with high functioning autism and were recruited via the parents’ association “Autismo Pisa APS” sited in Pisa. TD were recruited in primary schools in Parma as children matching the same age criterion used for the ASDs group. Children with a history of neurological or psychiatric disorders were not enrolled, as well as those whose parents or teachers expressed concerns about their development.

Informed written consent was obtained from the parents of all participants, and oral consent from each child. This study was approved by the Local Ethical Committee (Comitato Etico Area Vasta Emilia Nord, prot.n.13051) and was conducted according to the Helsinki Declaration.

### 2.2. Procedures

#### 2.2.1. Neuropsychological Evaluation

All children were evaluated with a neuropsychological battery including social perception and visuo-spatial domains. Both of them were indexed by subscales of the NEPSY-II test [27].

#### 2.2.2. Social Perception

Social perception domain is evaluated by two different subtests of NEPSY-II [27]: theory of mind and affect recognition. Each subtest is designed to measure a different set of skills necessary for understanding the feelings, perceptions, and intentions of others.

Theory of mind (ToM) includes two tasks. In the verbal task, scenarios or pictures are shown to the child, who has later to answer questions that require knowledge of another individual’s perspective. In the contextual task, the child is shown a picture depicting a social situation in which the face of the target individual is not shown. The child is then asked to select one out of four photographs, as the one depicting the most appropriate affect for the target individual in the picture. Overall, theory of mind evaluates the ability to comprehend others’ perspectives, intentions, and beliefs.

The affect recognition (AR) test assesses the ability to recognize affects (happiness, sadness, anger, fear, disgust, and neutral) from photographs of children’s faces in different tasks like same–different, on-line, and delayed similarity recognition.

#### 2.2.3. Visuo-Spatial Processing

The evaluation of the visuo-spatial domain was limited to the design copying (DC) test from NEPSY-II [27], aimed at assessing motor and visual-perceptual skills associated with the ability to copy two-dimensional geometric figures. It returns a global score (general score), as well as sub-scores measuring the degree of motor abilities, global attributes of the design, and local elements or details of the design. Taken together, these latter three sub-scores form the so-called design copying process score.

#### 2.2.4. Human Figure Drawing (HFD) Test

At the end of the neuropsychological evaluation, each participant was tested with the human figure drawing test, according to the procedures proposed by Royer [28]. HFD allows to explore the child’s level of intellectual maturity.

Each participant was given seven colored crayons (blue, green, red, yellow, purple, brown, and black), one pencil, one eraser, and 3 sheets of paper (21 × 29.5 cm) placed vertically. He was requested to draw, on the first sheet, a person. Upon completion of the first drawing, the participant was asked to indicate its gender, and the drawing was removed. Of note, most participants (36 out of 43) started from a male drawing. On the second sheet, the participant was then asked to draw a person of the opposite gender. Upon completion of the second human figure, the drawing was removed and a third sheet provided on which the child was invited to “draw a self-portrait”. Children were left free to choose the size and position in the sheet of their drawings. No time limit was imposed.

#### 2.2.5. Non-Human Figure Drawings

Subsequently, children were asked to accomplish a similar task, but depicting three houses (one house, another house, and their own one) rather than human figures. In this way, we obtained a second set of drawings allowing to test two interconnected hypotheses: first, whether children with ASD have peculiar features relative to TD, in particular in terms of similarity among drawings; second, whether such features are specific for human figures, or generalizable also to other subjects.

### 2.3. Coding of Drawings

The human figure drawings were scored according to the Maturity Scale [28]. This scale accounts for the presence of evolutionary details of the drawing, further grouped in the evaluation of head (23 items), body (32 items) and clothing (14 items), and color use. No Maturity Scale has been validated to date for houses, thus we limited its use to HFD.

To evaluate the differences among the human figure drawings, a similarity score was calculated [29]. The judgement of similarity was evaluated in parallel for three aspects, i.e., dimensions (height and width), body (shapes and colors), and attributes (shapes and colors of clothes and accessories). For each aspect, a score from zero (marked difference) to two (maximum similarity) was assigned. Such a score was evaluated among the three drawings, coupled two-by-two, thus obtaining 3 different scores. The similarity score was also calculated for the house drawings by adapting the scale of similarity used for the human figure drawings, and still comparing the houses to each other, two-by-two. Three different subscales (dimensions, attributes, and accessories) were considered.

We also calculated for the human figure drawings the value score [29]. This evaluates the presence of biases in the representational appearance between two drawings; for example, in the case of a colorful and richly decorated suit versus a monochromatic suit, the value score is greater for the former. The value score was evaluated in parallel for four aspects, i.e., the space occupied by each figure, the articulation of the body, the number of attributes, and the number of colors. These subscales were used to establish whether two figures were equally valued. For each subscale, scores from zero (equality) to two (marked or very marked difference) were assigned.

Two experimenters evaluated the drawings following strictly the guidelines indicated in Royer [28] for the maturity score, and in Bombi and Pinto [29] for the similarity and value scores. In the case of agreement between the two judgments, the agreed score was noted. Conversely, in the case of disagreement between the two experimenters, the final score was obtained after a joint evaluation among the two scorers and the senior author.

### 2.4. Statistical Analysis

All collected variables (see Table 1) underwent the Shapiro–Wilk’s W-test for verifying the assumption of normality. Parametric (one-way ANOVA, Bonferroni post-hoc) or non-parametric tests (Kruskal–Wallis, Mann–Whitney post-hoc) were applied accordingly. Eta-squared (η^2^) was calculated as a measure of effect size.

The factorial analysis was intended at verifying the homogeneity among groups in terms of age and non-verbal IQ. The same analysis on the neuropsychological scores and Maturity Scale aimed at evaluating the differences between groups.

Similarity and value scores spanned over a discrete and very narrow range (0, 1, 2), so a factorial analysis was not applicable. To evaluate comparatively these scores between groups, we then carried out a chi-squared tests inserting the count of each score for both ASD and TD, and evaluating whether their distribution varied across groups. In the case of a significant chi test, individual chi values were calculated to highlight which elements carried most of this disproportion.

Finally, we carried out correlation analyses testing the link between the Maturity Scale on one side, and indices of the social perception domain on the other. As we had no assumption on the linearity of such a relationship, we opted for using a Spearman rank correlation. In addition, the analysis was conducted separately for each group so to test the within-group dependency and not just a macroscopic between-group co-difference.

## 3. Results

**Figure 1:**
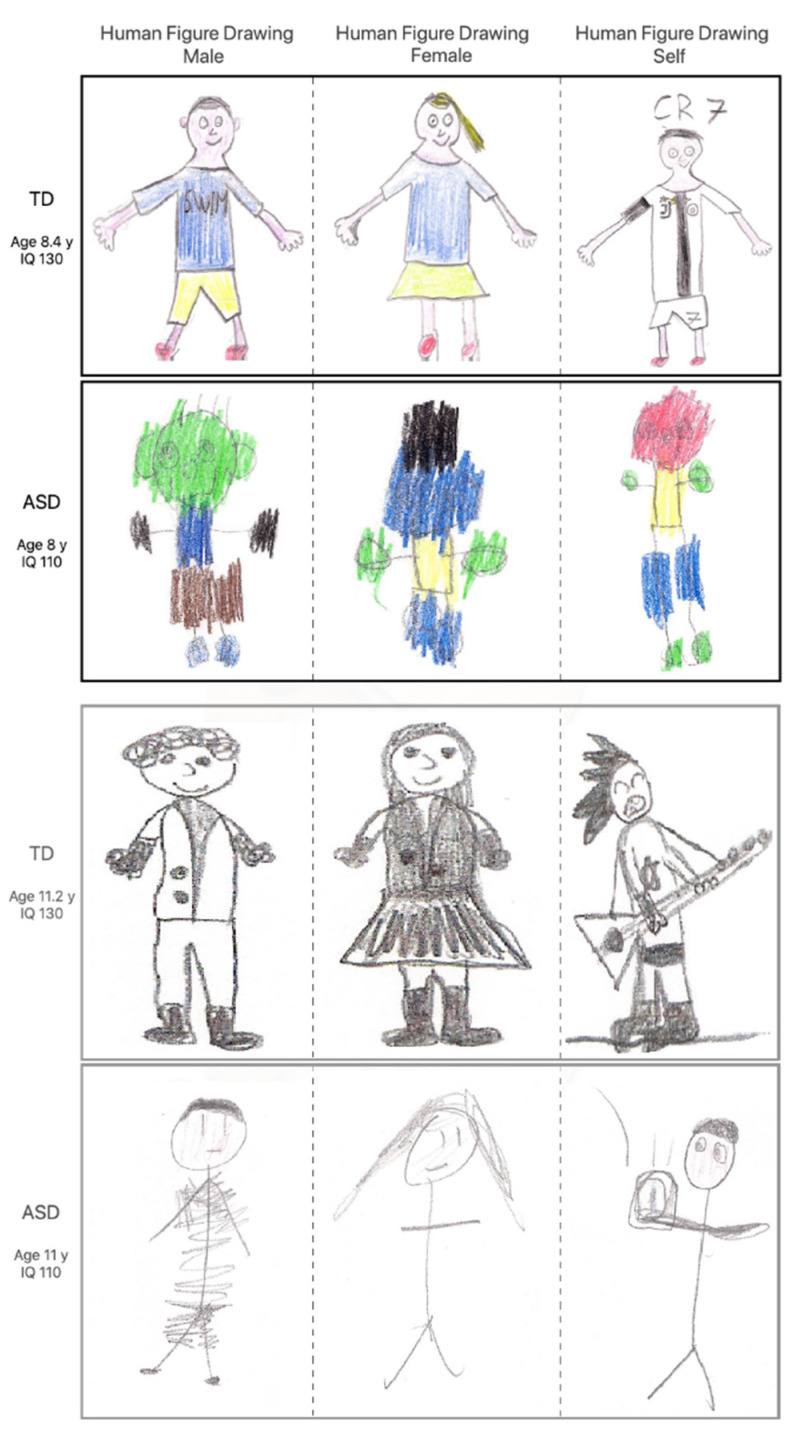
Examples of HFDs by ASD and TD children matched for age. Upper strips (black frame) relate to young children (8 years old), lower strips (grey frame) relate to older children in our range (11 years old). The triplets of maturity scores for the four subjects are (136.9, 125.0, 136.9), (72.4, 71.2, 76.2), (102.3, 112.1, 102.3), and (52.9, 50.1, 49.2), respectively.

Figure 1 reports the drawings from the HFDs of four participants, spanning over age (two 8 years old, two 11 years old) and groups (two TD and two ASDs).

We evaluated on the Maturity Scale and non-verbal IQ the presence of outliers as those subjects exceeding ±2 standard deviations of the sample’s mean. Two ASDs and one TD children were outliers for the human figure drawings Maturity Scale, while two children with ASDs were outliers for the non-verbal IQ scores. Then, the final sample consisted of 21 TD and 22 ASD children. Table 1 recaps the average scores for each test and group.

The matching between populations was witnessed by the lack of significant difference between groups, as returned by the Mann–Whitney test, in terms of age (U(22, 21) = 188, Z = 1.04, *p* = 0.30) and non-verbal IQ (U(22, 21) = 216.5, Z = −0.36, *p* = 0.72).

**Figure 2:**
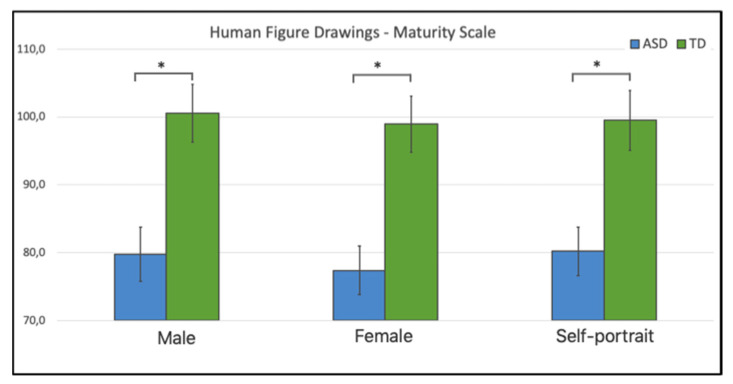
Maturity scale in the three drawings across ASDs and TD children. Bars indicate standard errors; asterisks a *p*-value < 0.01.

The Maturity Scale resulted as highly significant between groups, as by the one-way ANOVA, for each HDF (HDF-1, F(1, 41) = 12.67, *p* < 0.001, η^2^ = 0.23; HFD-2, F(1, 41) = 15.86, *p* < 0.001, η^2^ = 0.38; HFD-self, F(1, 41) = 11.73, *p* = 0.001, η^2^ = 0.22) (see Figure 2).

The same analysis returned a significant difference for ToM (F(1, 41) = 34.68, *p* < 0.001) indicating a marked difference between groups in the ability to comprehend others perspectives, intentions, and beliefs, with TD scores double relative to the ASD ones (TD M = 12.7, ASD M = 6.5). Similar results were obtained for the design copying general (DCG) score (F(1, 41) = 16.53, *p* < 0.001). When focusing on the subscale composing the design copy process (DCP), no difference between groups was found neither for DCP-global (TD M = 29.4, ASD M = 25.0) nor for DCP-local (TD M = 20.3, ASD M = 16.2). However, the motor subscale of copy design resulted to be significantly different between the two groups (DCP-motor: U(22, 21) = 127, Z = −2.51, *p* = 0.01) indicating that most of the difference in the DC performance between TD and ASDs relies on a poorer set of motor abilities of the latter group (TD M = 33.4, ASD M = 28.5). No difference emerges for scores at the AR test (U(22, 21) = 177, Z = −1.3, *p* = 0.19).

Concerning similarity, a chi-square test was applied to the three possible couples of human figure drawings (HFD-male vs. HFD-self, HFD-female vs. HFD-self, HFD-male vs. HFD-female). A significant difference emerged only in similarity between HFD-male and HFD-female (*χ*^2^ (2, N = 43) = 7.39, *p* = 0.02). Examining individual chi values, this significance appears mostly due to an over-presence of score 2 (maximum similarity) in ASDs (26 vs. 19.4 expected, *χ*^2^ = 2.2), while TD presented a lower rate of such a score (12 vs. 18.6 expected, *χ*^2^ = 2.3). No other HFD comparison turned out significant. The same procedure was applied to the similarity scores obtained for the houses, so to evaluate whether the tendency to hyper-similarity was specific for human figures, or generalizable also to other subjects. Of note, none of the three house comparisons returned significant results (all *p* > 0.4).

Despite the correlation analysis between the maturity at HFD and ToM scores resulted as not significant for both groups in all the three drawings, a different pattern emerged between TD and ASDs. Indeed, while TD showed a tendency towards significance in all drawings (all p around 0.08) and r coefficients around 0.4, ASDs fell apart from significance, with r values around 0.

Concerning the correlation between maturity and affect recognition, TD exhibited significant and positive findings for HFD-male and HFD-female, while no link was found for HDF-self. Of note, this pattern was fully reversed examining children with ASDs, who exhibited a significant correlation in HFD-self vs. AR, while non-significant results for HFD-male and HFD-female were found. The results of the correlation analysis are reported in Table 2.

## 4. Discussion

Tests based on human figure drawings have captured the attention of clinicians and psychologists for a long time, likely due to the easiness and ecologicity of their administration, with the aim to achieve a surrogate measurement of children intelligence [30]. However, a non-negligible body of literature has repeatedly challenged the validity of HFD tests [31,32], in particular when non-neurotypical individuals are investigated. The lack of firm guidelines about this matter, moreover, leads to inconsistencies in clinical neurodevelopmental practice, where HFD tests are treated with a relevance spanning over a spectrum from negligible to over-rated.

Pertaining to children with ASDs, although several researchers have suggested the utility of HFD tests in assessing this population [9,33], there are several well-known case studies in which children with autism accompanied by severe cognitive deficits have exhibited a superior drawing ability [34,35]. The findings of the present study align with the view that ASDs have lower maturity in drawings than TD. In line with the revision of the DAMT by Harris (1963) [3], we requested participants to draw three different human figures, yet no substantial differences appeared across these, suggesting a globally lower functioning in drawing rather than a hallmark specific for a given subject (see for example [36]).

These findings suggest that the use of HFD tests with individuals with autism may be not warranted, failing to provide elements suited to drive clinical routines. Indeed, maturity scores are obtained via a normalization against a TD normative population which has—on average—higher scores. To be usable and informative at the individual level, data from a normative population including only children with ASDs should be collected, or at least reference values should be provided to clinicians about the range of maturity scores specific for autistic individuals.

Once ascertained that to date maturity cannot inform about an individual child with ASDs, it is still possible that scores at HFD correlate with the functioning of specific domains. This is why we tested in the same participants the construct of social perception that reflects a variety of psychological processes, mostly involved in ASD symptomatology, i.e., ToM and AR. ToM is defined as the cognitive ability leading to the awareness that others have minds with mental states, information, and motivations that may differ from one’s own, allowing an individual to cognitively explain others behaviors [37]. In our study, coherently with previous literature [see 17], ToM scores resulted as highly segregated between ASDs and TD, yet no correlation emerged within both groups with HFD performance. This suggests a certain independency between performance in HFD and ToM tests, in turn reflecting a segregation between the underlying functions.

Concerning affective recognition, a completely different pattern was highlighted. Indeed, no difference appeared at the factorial analysis, suggesting a globally preserved (or restored via a rehabilitation intervention) ability of children with ASDs to explicitly recognize emotional expressions relative to control peers. Our results are in line with Narzisi et al., [38] who found that children with ASDs performed equally well as TD children in the affect recognition test (see also [39]), and with Tracy et al., [40], in which participants with ASDs succeeded in deciding whether an emotional photograph matched a target emotion. However, there is some evidence of atypical face perception processes in individuals with ASDs [41,42]. Indeed, while a deficit in face processing is not pathognomonic (meaning that it is not a definitive diagnostic sign), many children show a significant impairment in encoding facial features. In addition, Kuusikko et al. [21] indicated that despite that emotion recognition may improve with age in children with ASDs, it never completely achieves the level of typically developing individuals. Difficulties with social cognition do not always emerge in structured test situations, especially in high-functioning individuals with ASDs, and this may explain these inconsistent results. According to Harms et al., [43], some individuals with ASDs may utilize compensatory mechanisms (such as explicit cognitive processes) making the performance on facial emotion recognition tasks adequate in test situations, while atypical processing of stimuli is shown in neuroimaging studies [44] and difficulties in emotion recognition in real-life situations [45] are well-known.

Beyond the factorial analysis, we conducted a correlation analysis between scores obtained in HFD in the Maturity Scale and those obtained in the affective recognition test for both groups in order to get insights on the possible link between emotion recognition and drawing performance across multiple to-be-drawn subjects. Our results indicated not only the presence of positive and significant correlations, but also that they largely differed between groups. TD children showed a maturity score significantly associated with affect recognition in drawing other individuals, but not in self-portraits. An opposite pattern emerged for children with ASDs, whose sole maturity in self-portraits significantly correlated with the affective recognition scores.

Such a result could be interpreted in a very fascinating manner: in TD children, the capability to recognize others’ emotions could underlie the competence to graphically represent others. In turns, in children with ASDs, emotion recognition is not associated with maturity in the representation of others, but rather it correlates only with maturity in the self-representation. In other words, affective recognition skills might be used by TD to decode the outer social world, but by ASD only to express/represent themselves.

One could speculate that this selective link reflects a lack of experience of the social world in ASD [10], due to their socio-communicative atypicalities and relational poverty further impacting on their cognitive style [46]. The link between maturity and social communicative skills is very interdependent in children with ASDs and it has been the subject of extensive theoretical and empirical investigations [47]. In children with TD, the goodness of early social experiences has been linked to later maturity outcomes [48,49]. As reported by Vivanti (2016) [47], on the contrary, it is plausible that an altered sociability during early development may uniquely impact social dimensions of learning, including de facto the ingredients underlying affective recognition.

Despite only partially covering the abilities underlying the human figure drawing test, we evaluated the design copying test from NEPSY-II [27] to collect an index of an overall drawing performance, comparative between the two groups. The factorial analysis showed a significant difference between children with ASDs and TD children with a difficulty in the global ability to copy two-dimensional geometric figures for the first group, suggesting a weakness in visuomotor integration. The global ability to copy is generally associated with preserved visual-perceptual skills, and our findings are consistent with the validation study of the NEPSY-II by Korkman et al. [27] and with the more recent study by Narzisi et al. [38]. A similar difference was found for the design copy process, and this effect resulted mainly due to one of its subscales, namely the motor subtest. Further, in this case, the group with ASD presented difficulties in fine motor control, in line with a large body of literature pointing at motor disorganization as one of the determinant features in autism [50].

## 5. Conclusions

In conclusion, our study indicates that children with ASDs have a marked deficit in the human figure drawing test. The lack of a global consensus in the interpretation of HFD results, as well as of normative data peculiar for autism, advocate for an effort, joint by developmental psychology and neuropsychiatry, to achieve a reliable framework for the assessment of individuals with ASDs.

However, out of the clinical practice, tests based on HFD could be considered in basic research for revealing dependencies between drawing performance and neuropsychological features, thus possibly providing hints on the functioning of autism. We hereby showed the existence of a link between maturity in HFD and affect recognition, but the specificity of this result for autism across neurodevelopmental disorders as well as the neural mechanisms at the basis of this alteration have yet to be ascertained. To answer these points, future studies might consider to test additional groups of patients, possibly involving affective disturbances, and accompanying HFD with neurophysiological recordings.

## Figures and Tables

**Table 1:** Main scores for each test and group. Each cell contains the average score (M) and its standard deviation (SD).

					Design Copying Process (DCP)		
	Non-Verbal IQ	Theory of Mind (ToM)	Affect Recognition (AR)	Design Coping (General- DCG)	Motor Ability (DCP-M)	Global Attributes (DCP-Glob)	Local Elements (DCP-Loc)
**TD**	M = 113.3	M = 12.7	M = 9.4	M = 9.4	M = 33.4	M = 29.4	M = 20.3
	SD = 13.2	SD = 2.9	SD = 3.3	SD = 2.4	SD = 4.4	SD = 5.1	SD = 4.5
**ASD**	M = 112.3	M = 6.5	M = 7.8	M = 5.8	M = 28.5	M = 25.1	M = 16.2
	SD = 11.1	SD = 3.9	SD = 4.3	SD = 3.3	SD = 7.1	SD = 8.2	SD = 6.6

**Table 2:** Spearman correlation analysis between maturity and affect recognition scores, reporting (*r*) and *p*-values in the TD and ASD groups. Significant correlations (*p* < 0.05) are highlighted in grey.

Group	HFD	*r* (Spearman)	*p*-Value
**TD**	Male	0.53	0.015
	Female	0.53	0.012
	Self	0.33	0.14
**ASD**	Male	0.20	0.36
	Female	0.32	0.13
	Self	0.45	0.03
